# Diagnostic Performance of Frequency-Domain Optical Coherence Tomography to Predict Functionally Significant Left Main Coronary Artery Stenosis

**DOI:** 10.1155/2021/7108284

**Published:** 2021-11-15

**Authors:** Konstantina P. Bouki, Delia I. Vlad, Nikolaos Goulas, Vaia A. Lambadiari, George D. Dimitriadis, Athanasios A. Kotsakis, Kyriaki Barοutsi, Konstantinos P. Toutouzas

**Affiliations:** ^1^2^nd^ Department of Cardiology, General Hospital of Nikea-Piraeus, Nikaia, Greece; ^2^2^nd^ Department of Internal Medicine, University of Athens, Attikon Hospital, Athens, Greece; ^3^Department of Medical Imaging, General Hospital of Nikea-Piraeus, Nikaia, Greece; ^4^1^st^ Department of Cardiology, University of Athens, Hippokration Hospital, Athens, Greece

## Abstract

**Aims:**

The aim of this study was to assess the safety and diagnostic efficacy of frequency-domain optical coherence tomography (FD-OCT) in identifying functional severity of the left main coronary artery (LM) stenosis determined by fractional flow reserve (FFR).

**Methods and Results:**

101 patients with LM lesion (20–70% diameter stenosis angiographically) underwent FFR measurement and FD-OCT imaging of the LM. The following parameters were measured by FD-OCT in the LM: reference lumen area (RLA), reference lumen diameter (RLD), minimum lumen area (MLA), minimum lumen diameter (MLD), % lumen area stenosis, and % diameter stenosis. The LM lesions were analyzable by FD-OCT in 88/101 (87.1%) patients. FFR at maximum hyperemia was ≤0.80 in 39/88 (44.3%) patients. FFR values were correlated significantly with FD-OCT-derived LM lumen parameters. An MLA cutoff value of 5.38 mm^2^ had the highest sensitivity and specificity of 82% and 81%, respectively, followed by an MLD of 2.43 mm (sensitivity 77%, specificity 72%) and AS of 60% (sensitivity 72%, specificity 72%) for predicting FFR <0.80.

**Conclusions:**

FD-OCT is a safe and feasible imaging technique for the assessment of LM stenosis. An FD-OCT-derived MLA of ≤5.38 mm^2^ strongly predicts the functional severity of an LM lesion.

## 1. Introduction

Significant left main (LM) coronary artery disease (CAD) has been considered a determinant of increased cardiac mortality approaching 50% at 3-year follow-up [1]. Because of its clinical significance, the accurate assessment of the severity of an LM lesion is very important. Although coronary angiography has been accepted as the gold standard for the evaluation of CAD, the severity of an LM stenosis is often underestimated or overestimated [2, 3]. Proximal location of the lesion, vessel tortuosity, overlap, or foreshortening are common limitations of the coronary angiography for the quantitative analysis of the LM stenosis [2, 3]. In an effort to improve our diagnostic accuracy for the evaluation of LM disease, several techniques have been used.

Fractional flow reserve (FFR) is the current standard method for the functional assessment of a coronary lesion severity. It has been shown that an FFR value >0.80 predicts a positive outcome for patients with LM disease and can be used as an accurate and safe criterion for postponing revascularization [3, 4]. However, an important limitation of the LM FFR is the confounding effect of downstream stenosis which are often present in patients with LM disease.

Intravascular ultrasound (IVUS) has also been used in LM lesion assessment. A multicenter prospective study showed that a minimum lumen area (MLA) >6 mm^2^ is a safe criterion to defer revascularization, while several other IVUS studies [5–7] have proposed different cutoff values ranging from 4.5 mm^2^ to 7.5 mm^2^.

Fourier-Domain Optical Coherence Tomography (FD-OCT) provides high-resolution images of the coronary arteries allowing superior lumen border detection compared to IVUS [8–12]. The possibility of imaging the LM using such a high-resolution imaging modality as FD-OCT is attractive [13]. However, FD-OCT has the disadvantage of the need for full blood displacement by contrast injection during image acquisition, making difficult the imaging of large vessels with proximal location such as LM. Recently, Burzotta et al. [14, 15] showed that FD-OCT assessment of nonostial segment of the LM is feasible. To date, no study has evaluated the use of this imaging technique in the assessment of LM lesions in correlation with the FFR.

The purpose of the present study was to assess the safety and diagnostic efficacy of FD-OCT in identifying functional severity of the LM stenosis determined by FFR.

## 2. Methods

### 2.1. Study Population

From May 2015 to January 2020, all patients who underwent coronary angiography in General Hospital of Nikea and were found to have isolated LM stenosis (20%–70% diameter stenosis angiographically) were prospectively enrolled in the study. In all of these patients, FFR measurement and FD-OCT imaging of the LM before any intervention were attempted. Exclusion criteria were as follows: patients with LM stenosis >70% or <20%, patients with significant distal disease (left anterior descending artery (LAD) or left circumflex artery (LCX) stenosis ≥50%), acute myocardial infarction, abnormal regional wall motion of the left ventricle, chronic kidney disease (serum creatinine >1.5 mg/dL), congestive heart failure, and known malignant disease. All demographic and clinical data were collected prospectively.

All patients were informed, and written consent was obtained for every patient.

### 2.2. Cardiac Catheterization Procedure

Coronary angiography was performed with the standard technique through the femoral or the radial artery approaches, according to the operator's preference, using 6 Fr guiding catheters. All patients received 5.000–7.500 IU of unfractionated heparin and intracoronary isosorbide dinitrate (0.2-0.3 mg) before angiography.

### 2.3. FFR Measurement

Equalization was performed when the guidewire sensor was positioned at the tip of the guiding catheter. After the equalization, a 0.014-inch pressure guidewire (St. Jude Medical, USA) was positioned ≥3 cm distal to the LM in either the LAD, LCX, or both, depending on which artery was least diseased distally. The FFR was measured during maximal hyperemia induced by intravenous infusion of adenosine at 140–280 *μ*g/kg/min [7]. In patients with LM proximal stenosis, the FFR measurement was made with the guiding catheter out of the LM. Stenosis was labeled as functionally significant if FFR ≤0,80.

### 2.4. FD-OCT Imaging

After FFR assessment, FD-OCT imaging was performed using frequency-domain imaging system (C7, St. Jude Medical, USA). 200 *μ*g of intracoronary nitroglycerin was administered before FD-OCT imaging to avoid coronary spasm. The LM was cannulated with a 6Fr extra backup guiding catheter without side holes and attention was paid to be in good alignment with the vessel. A 2.7 Fr FD-OCT imaging catheter (Dragonfly, St. Jude Medical, USA) was advanced over a conventional angioplasty guidewire distal to the LM bifurcation. FD-OCT pullbacks were attempted from LAD or LCX according to the operator's decision. Image acquisition was performed by an automated pullback with a speed of 20 mm/sec, while the blood was removed by continuous manual injection of iso-osmolar contrast (Iodixanol 370, Visipaque, GE Healthcare, Ireland) through the guiding catheter. When the lesion was in the proximal part of the LM, to avoid the risk of masking, the guiding catheter was positioned just in front of the LM ostium or slightly disengaged during imaging acquisition. The FD-OCT pullbacks were repeated until adequate visualization allowing quantitative assessment of the whole LM segment was obtained. The required number of pullbacks and the amount of injected contrast were calculated. All images were stored digitally and analyzed offline by the FD-OCT console. The acquisition run with the best image quality was used for the offline analysis.

### 2.5. Angiographic Analysis

**Figure 1:**
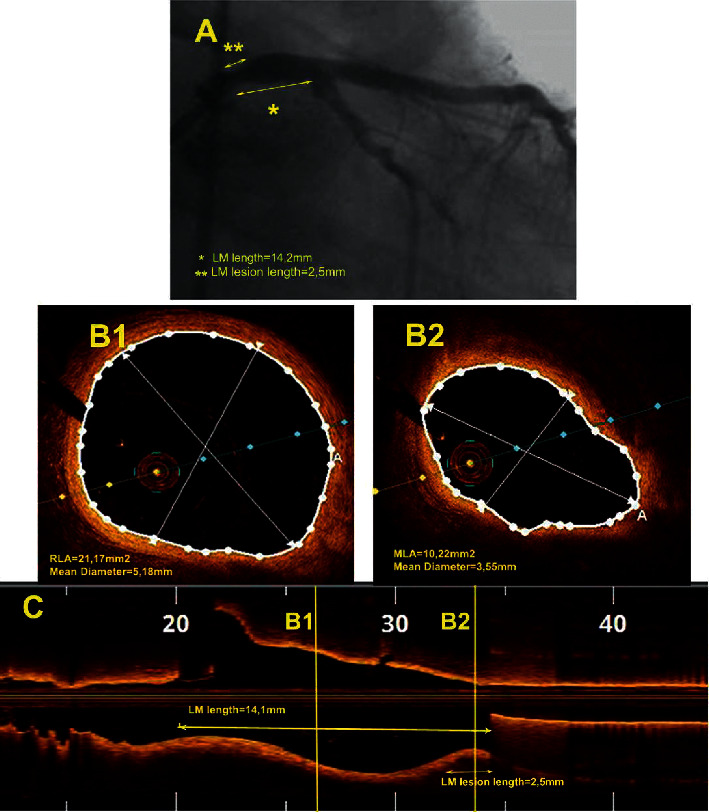
Example of proximal left main (LM) stenosis. (a) Angiographic view showing a proximal LM stenosis. The measurements of the LM length and the lesion length are also presented (double arrows). (b). Optical coherence tomography (FD-OCT) cross-sectional images of the LM with measured lumen dimensions. B1: reference lumen area (RLA); B2: minimum lumen area (MLA). (c) Longitudinal FD-OCT reconstruction of the LM showing the location of measurements (B1 and B2) and the measurements of the total LM length and the lesion length (double arrows).

All angiograms were analyzed by an angiographer who was blinded to the clinical and FD-OCT findings, using quantitative coronary angiographic (QCA) measurements. Quantitative coronary angiography (QCA) was performed offline by a skilled analyzer using standard commercial software (CAAS QCA 5, Pie Medical, Maastricht, Netherlands). The LM was divided into 3 segments: the proximal 1/3 of the LM, the mid 1/3 of the LM, and the distal 1/3 of the LM. LM lesions were characterized as proximal if they were located in the proximal part of the LM. Reference lumen diameter (RLD), minimum lumen diameter (MLD), percent diameter stenosis (% DS), length of the whole LM (from the aortic orifice till the bifurcation of LAD and CX), and LM lesion length were determined by QCA (Figure 1(a)). The QCA analysis was conducted from the single-best-available projection with the least foreshortening and the most severe stenosis.

### 2.6. FD-OCT Imaging Analysis and Measurements

FD-OCT images analysis was performed according to the criteria of the International Working Group for Intravascular Optical Coherence Tomography Consensus Standards for Acquisition, Measurement, and Reporting of Intravascular Optical Coherence Tomography studies [16, 17]. All FD-OCT images were analyzed by an experienced analyst who was blinded to the angiographic and FFR results. For the present study, quantitative FD-OCT analysis focused on the whole LM region from the catheter tip until the ostia of its bifurcation branches (defined as the first cross-sectional image of the daughter vessel where the other branch was not visible). The LM was divided into 3 segments, proximal, mid, and distal, in accordance with the angiographic analysis. The following parameters were measured from the cross-sectional images: reference lumen area (RLA), minimum lumen area (MLA), percent area stenosis (% AS), RLD, MLD, and % DS (Figure 1(b)). The length of the LM as well as the LM lesion length was measured from the long-axis view (Figure 1(c)).

Frames with lumen border visibility less than 270° were considered artifact and excluded from the analysis. Causes for artifact frames were recorded and classified as follows: portion of the image out of screen (because of eccentric position of the FD-OCT catheter or very large size of the LM) and inadequate blood clearance of the lumen. Acquisition runs with many artifact frames causing inability of measurements were excluded from the analysis.

An LM proximal lesion was considered analyzable (visible and measurable) by FD-OCT if 2 conditions were satisfied:

1. The total length of the LM measured by FD-OCT was equal to the total length of the LM measured by angiography (differences smaller than 1 mm were considered negligible)
2. The visualization of the proximal part of the LM was optimal with less than 5 artifact frames

In accordance to the above analysis, lesions located at the mid or distal part of the LM were considered analyzable by FD-OCT if the visualization was optimal at the mid or distal segment of the LM with less than 5 artifact frames at each part.

### 2.7. Statistical Analysis

Data were analyzed using IBM SPSS Statistics version 23. Categorical variables were presented as counts and percentages. Normally distributed continuous variables were presented as mean values with standard deviations; Shapiro-Wilk test was used to determine whether data were normally distributed. Comparisons between categorical variables were done with *x*^2^ (Pearson's chi-square test). Comparisons between study groups were performed with *t*-test and correlations were tested by the Pearson correlation coefficient. Linear regression analysis was used to determine the correlation coefficients between FFR and FD-OCT measurements and presented using scatter plot graphics. Receiver operating characteristic (ROC) curve analyses of MLA, MLD, and % AS in predicting a positive FFR (≤0.80) were performed. The area under the curve (AUC) of the ROC curves was estimated and used as the index of classification accuracy. Values of *p* < 0.05 were considered statistically significant.

## 3. Results

### 3.1. Baseline Clinical and Angiographic Characteristics

A total of 128 patients were included in the study. In 18 patients, the operator did not perform FD-OCT imaging on the basis of anatomical characteristics, duration of the procedure, or patient discomfort. 9 patients out of 128 (7%) were excluded because of inadequate quality of FD-OCT images. Finally, 101 patients (60 male and 41 female) were prospectively enrolled in the study. Baseline clinical and angiographic characteristics of the patients are shown in Table 1. Among the 101 patients, 30 (29.7%) had a proximal lesion of the LM and 71 (70.3%) at the mid or distal segment. Most of the patients had de novo lesion of the LM (99/101 (98.0%) patients), with a mean % diameter stenosis of 45.74 ± 11.3 by QCA. For the total of 101 lesions, the mean FFR value was 0.83 ± 0.07. 42 out of 101 (41.5%) patients had an FFR ≤0.80 (ischemic group). The ischemic group was found to have more severe LM stenosis (as it was expected) and higher incidence of old myocardial infarction (Table 1). ROC analysis showed that a cutoff value of 50% angiographic stenosis by QCA could predict ischemic FFR with a sensitivity of 69.2% and a specificity of 65.3% (AUC = 0.66, 95% CI = 0.54–0.78, *p*=0.007).

### 3.2. FD-OCT Imaging Procedural Characteristics: Comparison between Angiographic and FD-OCT Measurements

FD-OCT pullbacks were performed from LAD in 76 patients and from LCX in 25 patients. During flushing, 6 patients described chest pain. No patient had arrhythmias, cardiac biomarker elevation, or contrast induced nephropathy.

The LM lesions were analyzable by FD-OCT in all patients (71/71 patients, (100%)) with mid or distal location of the lesion. However, in patients with proximal location, the lesion was analyzable by FD-OCT only in 17/30 (56.4%) patients. Subsequently, the final FD-OCT measurements were available in 88/101 (87.12%) patients. The deep position of the guiding catheter into the LM was the most common reason for the nonanalyzable LM proximal lesions (9/13 cases (69.3%)), while the large number of artifact frames was a second reason (4/13 cases (30.7%)). In patients with proximal LM lesion, the total number of pullback runs (4.53 ± 0.81 versus 2.44 ± 1.06, *p* < 0.001, respectively) and the related contrast infused (47.43 ± 6.70 versus 26.28 ± 9.63, *p* < 0.001, respectively) were significantly higher than those in the patients with mid or distal LM stenosis.

There was a significant correlation between QCA and FD-OCT measurements of the LM length (*r* = 0.698, *p* < 0.001), RLD (*r* = 0.524, *p* < 0.001), MLD (*r* = 0.360, *p*=0.001), and degree of % DS (*r* = 0.374, *p* < 0.001). However, FD-OCT measured larger RLD, MLD, smaller % DS, and shorter LM length compared to QCA (Table 2).

### 3.3. Relation between FD-OCT Measurements and FFR

Table 3 shows the comparison of FD-OCT measurements between the ischemic (FFR ≤0.80) and the nonischemic (FFR >0.80) groups of patients. Among the 88 patients with analyzable lesions by FD-OCT, 39 patients (44.3%) had significant stenosis based on FFR values (FFR of ≤0.80 at maximum hyperemia) (Table 3). These lesions had longer lesion length, smaller RLA, smaller MLA, greater % AS, smaller MLD, and greater % DS by FD-OCT compared with the lesions with an FFR >0.80 (Table 3).

**Figure 2:**
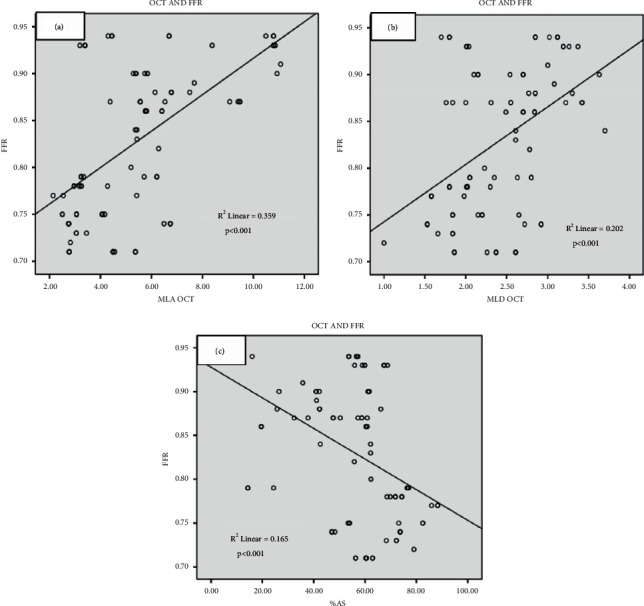
Relation between optical coherence tomography (OCT) measurements and fractional flow reserve (FFR). (a) Relation between minimum lumen area (MLA) and FFR, (b) relation between minimum lumen diameter (MLD) and FFR, and (c) relation between percent area stenosis (% AS) and FFR.

There was a significant correlation between FFR values and FD-OCT measurements of the MLA (*R*^2^ = 0.359, *p* < 0.001), MLD (*R*^2^ = 0.202, *p* < 0.001), and % AS (*R*^2^ = 0.165, *p* < 0.001) (Figure 2).

**Figure 3:**
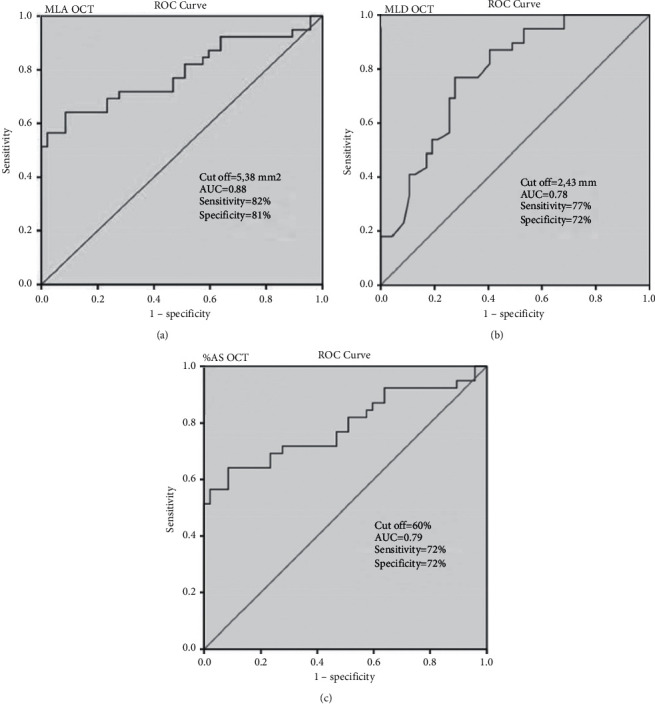
Receiver operating characteristic (ROC) curves. (a–c) ROC curves for OCT-derived MLA, MLD, and % AS to predict FFR ≤0.80. The abbreviations are as in Figure 2.

Receiver operating characteristic curves for FD-OCT-derived MLA, MLD, and % AS were used to predict functionally significant LM stenosis (Figure 3). An MLA cutoff value of 5,38 mm^2^ had the highest sensitivity and specificity of 82% and 81%, respectively (AUC = 0.88, 95% CI: 0.80–0.95, *p* < 0.001), followed by an MLD of 2.43 mm (sensitivity 77%, specificity 72%, AUC = 0.78, 95% CI: 0.68–0.88, *p*=0.001) and % AS of 60% (sensitivity 72%, specificity 72%, AUC = 0.79, 95% CI: 0.69–0.89, *p* < 0.001) (Figure 3).

**Figure 4:**
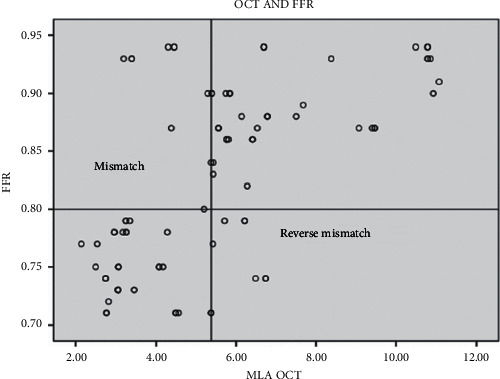
Scatter plot of FD-OCT MLA versus FFR values. The abbreviations are as in Figure 2.

Among 41 lesions with an MLA of ≤5.38 mm^2^, 9 (22.0%) lesions had an FFR >0.80 (mismatch), while among 45 lesions with an MLA >5.38 mm^2^, only 7 (15.5%) had an FFR ≤0.80 (reverse mismatch) (Figure 4). Additionally, taking a cutoff value of MLA ≤3.20 mm^2^ for prediction of ischemic FFR, only 1 out of 17 lesions (5.6%) with MLA ≤3.20 mm^2^ had an FFR >0.80 (mismatch 5.6%). Meanwhile taking a cutoff value of MLA >6.76 mm^2^ for prediction of nonischemic FFR, 0 out of 39 lesions (0%) with MLA >6.76 mm^2^ had an FFR ≤0.80 (reverse mismatch 0%).

Comparison between QCA (AUC = 0.66, 95% CI = 0.54–0.78, *p*=0.007) and FD-OCT for the prediction of ischemic FFR (AUC = 0.88, 95% CI: 0.80–0.95, *p* < 0.001) showed significant superiority of FD-OCT for area under the ROC curve (McNemar *p*=0.013).

### 3.4. Clinical Outcomes

There were no complications during the diagnostic procedures. All the 42 patients in whom the FFR of the LM was ≤0.80 underwent revascularization with PCI successfully. No event occurred during hospitalization.

## 4. Discussion

In the present study, we demonstrated that FD-OCT was safe and feasible for the evaluation of the LM lesions except the proximal stenosis which were analyzable only in 56% of cases. We also found that there was a strong correlation between FD-OCT-derived MLA, MLD, and % AS with FFR measurements. Among the different measured lumen parameters, MLA cutoff value of 5,38 mm^2^ provided the best sensitivity and specificity to predict the functional severity of the LM stenosis (82% and 81%, respectively, AUC = 0.88).

There are few studies [13, 14, 18, 19] evaluating the accuracy of FD-OCT for the assessment of LM and there are even less concerning the ability of FD-OCT to image the proximal LM part [13, 19]. Burzotta et al. [14] excluded the ostial LM lesions and found that the LM bifurcation can be perfectly evaluated by FD-OCT. Fujino et al. [19] confirmed that FD-OCT assessment of the LM is feasible but the LM ostium was properly imaged only in 12.5% of patients. However, this study was a retrospective small study which was not dedicated to evaluating the LM by FD-OCT. Recently, Roule et al. [13] found that overall more than 90% of the quadrants of the LM were adequately assessable by FD-OCT, while most artifacts (18.6%) were located at the proximal part of the LM. The present study confirmed the difficulty of FD-OCT to evaluate the proximal part of the LM as we found that only half (56%) of the proximal LM lesions were analyzable by FD-OCT. However, this proportion was much higher than that suggested by previous studies. It is worth mentioning that our study is the first prospective study dedicated to evaluating the ability of OCT for the LM imaging. We used for the first time 2 predefined criteria for the LM visibility by FD-OCT: (1) the number of FD-OCT artifacts frames in LM should be less than 5 and (2) the total length of the LM measured by FD-OCT should be equal to the angiographic LM length. Consequently, the higher proportion of FD-OCT analyzable lesions at the proximal LM we found may be due to our different methodology. In this study guide, extension catheter was not used during FD-OCT imaging. According to some reports, the use of this catheter might have improved the imaging of LM proximal lesions. However, this remains to be tested in future studies.

There are no data in the literature regarding FD-OCT-derived lumen parameters to predict the physiologic significance of an LM stenosis. However, the technique has already been used to estimate the functional severity of coronary artery stenosis excluding LM lesions. In particular, Gonzalo et al. [20] found an FD-OCT-derived MLA <1.95 mm^2^ as the best cutoff value to predict FFR <0.80 with a sensitivity of 83% and a specificity of 63%. Meanwhile, later, Dato et al. [21] using a combination of FD-OCT-derived parameters (% AS ≥70%, MLA <2,5 mm^2^, and plaque ulceration) demonstrated higher diagnostic accuracy with a sensitivity of 91% and a specificity of 93% for the prediction of an FFR <0.80 in non-LM coronary artery lesions [15]. The same researchers more recently suggested that, in patients with intermediate distal LM disease, combination of different FD-OCT-derived parameters has the potential to select those patients in which revascularization can safely be deferred [21]. However, the value of FD-OCT in the estimation of the functional severity of LM lesions remains unknown.

IVUS which is an older but well-established intracoronary imaging technique has been used in several studies to evaluate the severity of the LM disease. Traditionally, an IVUS-derived MLA <6.0 mm^2^ is considered to represent functionally significant LM stenosis. This strategy is based on data from a number of observational studies. Jasti et al. found that an IVUS-derived MLA <5.9 mm^2^ strongly predicts the physiological significance of an LM coronary stenosis [22]. In LITRO, a multicenter, prospective study [6], it was demonstrated that it is safe to defer revascularization if the IVUS-derived MLA was ≥6 mm^2^. Smaller cutoff values of IVUS-derived MLA have been found in Asian patients with generally smaller heart sizes [5, 7]. These studies have suggested that an IVUS-derived MLA of 4.5–4.8 mm^2^ may be the most appropriate.

The LM MLA cutoff value of 5.38 mm^2^ identified by FD-OCT in our study was lower than IVUS-derived MLA of 6.0 mm^2^ used in current practice. However, this is in accordance with observations from previous studies [23–25] which have shown that FD-OCT estimates smaller vessel MLA compared to IVUS and the size of this discrepancy is approximately 10%. Notably, the LM MLA cutoff value we found by FD-OCT was also 10% lower than that of IVUS. According to Bezerra et al. [25], possible explanations for this size difference between FD-OCT and IVUS may be the following: (1) better lumen discrimination by FD-OCT may allow more accurate lumen visualization than IVUS, (2) faster pullback of FD-OCT catheter may preclude selection of frames at maximum diastole, and (3) the smaller profile of the FD-OCT catheter compared to IVUS may cause less stretch (Dotter effect) of the vessel in severe stenosis.

Considering the prognostic impact of the identification of significant LM stenosis and because the MLA cutoff value in our study showed a 25.6% rate of mismatch and 19.0% rate of reverse mismatch, the decision-making cannot be relied on an FD-OCT MLA alone. Until now, the FFR has been the gold standard in the evaluation of angiographic intermediate LM stenosis [26]. Therefore, FFR measurement for intermediate LM stenosis should be required to avoid unnecessary treatment. However, in cases of complex LM stenosis with additional significant disease in the LAD and LCX, in which FFR may underestimate the lesion, an FD-OCT MLA of 5.38 mm^2^ can help decision-making. Another issue that should be cleared is that imaging of the LM proximal located stenosis was suboptimal by FD-OCT in half of the cases in our study. Therefore, we do not support the use of this technique for imaging proximal LM lesions.

### 4.1. Study Limitations

This study is a single-center study with a relatively small sample size. We excluded patients with significant LAD or LCX stenosis which is a frequent problem in everyday practice. We did not assess the clinical value of the FD-OCT-derived MLA <5,38 mm^2^ in decision-making for revascularization. Larger-scale studies are warranted to confirm the presented data and moreover clinical follow-up study with the new FD-OCT criterion.

## 5. Conclusions

FD-OCT was safe and feasible for the evaluation of the LM lesions except the proximal LM lesions which were analyzable by FD-OCT in half of the cases. Among the analyzable LM lesions, an FD-OCT-derived MLA ≤5.38 mm^2^ was a useful criterion for the prediction of functional severity of an LM stenosis.

### 5.1. Impact on Daily Practice

FD-OCT is a safe and feasible imaging technique for the assessment of LM stenosis except the proximal stenosis which is visible and analyzable in only half of the cases. An FD-OCT-derived MLA ≤5.38 mm^2^ strongly predicts the functional severity of an LM lesion and can help towards the right clinical decision-making for the management of LM coronary artery disease.

## Figures and Tables

**Table 1:** Baseline clinical and angiographic characteristics of the study population.

	Patients (*n* = 101)	Patients FFR ≤0.80 (*n* = 42)	Patients FFR >0.80 (*n* = 59)	*p* value
*Baseline characteristics*				
Age (years)	63.18 ± 9.8	62.38 ± 9.8	63.75 ± 9.8	0.493
Male, *n* (%)	60 (59.4)	24 (57.1)	36 (61.0)	0.696
Hypertension, *n* (%)	56 (55.4)	19 (45.2)	37 (62.7)	0.082
Diabetes, *n* (%)	34 (33.7)	18 (42.9)	16 (27.1)	0.099
Dyslipidemia, *n* (%)	76 (75.2)	30 (71.4)	46 (78.0)	0.453
Current smoker, *n* (%)	59 (58.4)	22 (52.4)	37 (62.7)	0.299
Family history, *n* (%)	45 (44.6)	14 (33.3)	31 (52.5)	0.056
LV ejection fraction (%)	50.45 ± 9.0	50.71 ± 7.9	50.25 ± 9.9	0.803
Acute coronary syndrome, *n* (%)	32 (31.7)	14 (33.3)	18 (30.5)	0.961
Prior myocardial infarction, *n* (%)	9 (9.0)	5 (12.0)	4 (6.8)	0.187
Prior coronary intervention, *n* (%)	49 (48.5)	24 (57.1)	125 (42.4)	0.143
Proximal lesion LM, *n* (%)	30 (29.7)	14 (33.3)	16 (27.1)	0.501

*Angiographic characteristics*				
Total LM length, mm	14.23 ± 5.2	15.13 ± 5.4	13.59 ± 4.9	0.142
LM lesion length, mm	3.09 ± 1.4	3.57 ± 1.4	2.73 ± 1.3	0.003
LM reference lumen diameter, mm	3.89 ± 0.7	3.92 ± 0.7	3.87 ± 0.6	0.677
LM minimum lumen diameter, mm	2.09 ± 0.5	1.94 ± 0.5	2.19 ± 0.4	0.010
LM % diameter stenosis	45.74 ± 11.3	50.22 ± 10.7	42.56 ± 10.6	0.001
Mean FFR value	0.83 ± 0.07	0.75 ± 0.02	0.89 ± 0.03	<0.001

Values are presented as *n* (%) or mean ± standard deviation (SD). FFR = fractional flow reserve; LM = left main; LV = left ventricle.

**Table 2:** Angiographic and optical coherence tomography measurements of the LM lesions.

	QCA (*n* = 88)	FD-OCT (*n* = 88)	*p* value
LM length, mm	14.65 ± 5.3	12.48 ± 5.1	<0.001
Lesion length, mm	3.20 ± 1.5	3.72 ± 2.0	0.032
Reference lumen diameter, mm	3.79 ± 0.6	4.05 ± 0.6	<0.001
Minimum lumen diameter, mm	2.07 ± 0.5	2.46 ± 0.6	<0.001
Percent diameter stenosis (%)	44.78 ± 15.4	38.66 ± 14.4	<0.001
Reference lumen area, mm^2^	—	13.14 ± 4.1	
Minimum lumen area, mm^2^	—	5.82 ± 2.9	
Percent area stenosis (%)	—	55.04 ± 18.7	

Values are presented as *n* (%) or mean ± standard deviation. FD-OCT = frequency-domain optical coherence tomography; LM = left main; *n* = number of patients; QCA = quantitative coronary angiography.

**Table 3:** Comparison between optical coherence tomography measurements of LM stenosis with FFR ≤0.80 and FFR >0.80.

	FFR ≤0.80 (*n* = 39)	FFR >0.80 (*n* = 49)	*p* value
LM length, mm	13.75 ± 4.9	11.47 ± 5.0	0.035
Lesion length, mm	4.29 ± 2.5	3.24 ± 1.5	0.020
Reference lumen diameter, mm	3.94 ± 0.5	4.10 ± 0.7	0.128
Minimum lumen diameter, mm	2.11 ± 0.4	2.74 ± 0.7	<0.001
Percent diameter stenosis (%)	45.32 ± 14.1	33.35 ± 12.4	<0.001
Reference lumen area, mm^2^	11.86 ± 3.0	14.16 ± 4.6	0.008
Minimum lumen area, mm^2^	3.96 ± 1.3	7.31 ± 3.0	<0.001
Percent area stenosis (%)	64.42 ± 17.6	47.58 ± 16.3	<0.001

Values are presented as *n* (%) or mean ± standard deviation (SD). FFR = fractional flow reserve; LM = left main coronary artery; *n* = number of patients.

## Data Availability

The datasets used and/or analyzed during the current study are available from the corresponding author upon reasonable request.

## Abbreviations

AS: Area stenosis
AUC: Area under the curve
CAD: Coronary artery disease
CI: Confidence interval
DS: Diameter stenosis
FD-OCT: Frequency-domain optical coherence tomography
FFR: Fractional flow reserve
IVUS: Intravascular ultrasound
LAD: Left anterior descending artery
LCX: Left circumflex artery
LM: Left main coronary artery
MLA: Minimum lumen area
MLD: Minimum lumen diameter
OR: Odds ratio
QCA: Quantitative coronary angiogram
RLA: Reference lumen area
RLD: Reference lumen diameter
ROC: Receiver operating characteristic curve.
